# How to overcome inter-electrode variability and instability to quantify dissolved oxygen, Fe(II), mn(II), and S(−II) in undisturbed soils and sediments using voltammetry

**DOI:** 10.1186/1467-4866-13-6

**Published:** 2012-06-25

**Authors:** Aaron J Slowey, Mark Marvin-DiPasquale

**Affiliations:** 1 U.S. Geological Survey, Menlo Park, CA, USA; 2 Earth Sciences Division, Lawrence Berkeley National Laboratory, 1 Cyclotron Road, Mail Stop 90R1116, Berkeley, CA 94720, USA

## Abstract

**Background:**

Although uniquely capable of measuring multiple redox constituents nearly simultaneously with no or minimal sample pretreatment, voltammetry is currently underutilized in characterizing redox conditions in aquatic and terrestrial systems. Investigation of undisturbed media such as pore water requires a solid-state electrode, and such electrodes can be difficult to fabricate reproducibly. An approach to determine the concentrations of electroactive constituents using indirectly calibrated electrodes has been developed, but the protocol for and accuracy of this approach—the pilot ion method—has not been documented in detail.

**Results:**

A detailed procedure for testing electrode quality is provided, and the application and limitations of the pilot ion method have been documented. To quantify Fe(II) and Mn(II), subtraction of non-linear baseline functions from voltammetric signals produced better calibration curves than did linear baselines, enabled lower detection limits and reliable deconvolution of overlapping signals, and was successfully applied to sediment pore water signals. We observed that electrode sensitivities often vary by tens of percent, and that the sensitivity declines over time. The ratio of calibration slopes of Mn(II) to Fe(II) varied by no more than 11% from one Hg/Au electrode to another and Fe(II) concentrations predicted by the Mn(II) pilot ion were, on average, 13% different from their actual values. However, concentration predictions by the pilot ion method were worse for less than 15 μM Fe(II) (46% different on average). The ratio of calibration slopes of Mn(II) to S(−II) varied by almost 20% from one Hg/Au electrode to another, and S(−II) predicted concentrations were as much as 58% different from their actual values. These predictions of Fe(II) and S(−II) concentrations indicate that the accuracy of the pilot ion method depends on how independent calibration slope ratios are from the electrode used. At medium-to-high concentration for the ocean, naturally derived dissolved organic carbon did not significantly affect the baseline-corrected electrode response of Mn(II) and Fe(II), but did significantly affect the response of S(−II).

**Conclusions:**

Despite their intrinsic variability, Hg/Au electrodes fabricated by hand can be used to quantify O_2_, S(−II), Fe(II), and Mn(II) without calibrating every electrode for every constituent of interest. The pilot ion method can achieve accuracies to within 20% or less, provided that the underlying principle—the independence of slope ratios—is demonstrated for all voltammetric techniques used, and effects of the physicochemical properties of the system on voltammetric signals are addressed through baseline subtraction.

## Introduction

Reduction-oxidation (redox)-active species are integral to microbially mediated contaminant, nutrient, and carbon transformations in aquatic and terrestrial systems [1-6]. Electrochemical sensors have been routinely used over the past two decades to study redox processes and trace element speciation [7,8]. Among the electrochemical methods used for this purpose, voltammetry is able to analyze multiple dissolved redox constituents with no or minimum sample manipulation [9,10]. The pioneering work of Brendel and Luther [11] and Tercier and Buffle [10,12] in the development and use of microelectrodes has enabled *in situ* voltammetric analysis of redox constituents and trace metals in natural systems [13-17]. While progress has been made and researchers continue to voltammetrically analyze aquatic systems with microelectrodes, there is much room for growth in the number of users and deployments [18]. One impediment to growth is poor understanding of how to convert voltammetric signals to analyte concentrations. Although numerous papers show the quantitative results of such conversions, the process is not intuitive and could be explained in more detail and with specific application to natural samples. To help more environmental scientists use voltammetry to decipher microbial processes that mediate redox conditions in undisturbed soils and sediments, this paper shows how to quantify the voltammetric signals of O_2_, Mn(II), Fe(II), and S(−II) and overcome challenges imposed by the use of solid-state electrodes.

To achieve quantitative *in situ* measurements, one needs to overcome the imprecision of solid-state electrodes when fabricated and possible alteration of the sensors by chemical or biological agents, which cause initial differences in and instability of their analytical sensitivity. For reasons explained by Buffle and Tercier-Waeber [10], mercury (Hg) is the sensor material of choice for redox analysis of environmental systems. Hg-amalgam [11,19] or Hg-film [12,20-22] sensors are used to construct electrodes that meet additional requirements for *in situ* measurements such as insertion into porous media. The focus of this paper is on a 1 mm-diameter glass electrode equipped with a 100 μm Hg/Au amalgam sensor.

Once a Hg/Au amalgam electrode is made, there are at least three criteria by which to evaluate the quality of the Hg/Au amalgam sensor. First, in oxygenated solution, a Hg-based electrode should yield (a) an elongated, S-shaped current-potential relationship following O_2_ and H_2_O_2_ reduction and (b) alkali metal reduction at −1.7 V vs. Ag/AgCl or lower. When Au is plated well by Hg, alkali metal (typically Na^+^) reduction shifts to potentials well below −1.55 V such that a sufficient overpotential can be applied to measure Mn(II) [10,19,23]. The overpotential is defined as “the additional potential (beyond the thermodynamic requirement) needed to drive a reaction at a certain rate” [24].

The second criterion pertains to the current signal measured in a deaerated solution containing no electroactive constituents within the range over which Hg can be electrically polarized. At circumneutral pH, this range extends from about −0.05 V vs. Ag/AgCl, above which Hg is oxidized, down to about −1.7 V, below which alkali metals are reduced [10]. This range encompasses the redox potentials of O_2_, S(−II), Fe(II), and Mn(II). The electrode-water interface acts experimentally like a capacitor [24]. When polarized, an electrode surface accumulates charge and electrostatically retains an excess of aqueous cations or anions (the point of zero charge of a Hg electrode is around −0.5 V vs. Ag/AgCl [25,26]). A current flows during this process and is called the charging or capacitative current [9,24], which is measured continuously as the structure of the electrode-water interface evolves with the change in potential during a voltammetric scan. This capacitative current is also sometimes described as being nonfaradaic, in that it does not arise from electron transfer to or from an electroactive constituent. To quantify electroactive constituents, the faradaic signal needs to be isolated from the nonfaradaic signal. After each potential step or modulation in the voltammetric scan, both the capacitative and faradaic currents spike, but the capacitative current decays faster [10]. If scan rates are lower, the potentiostat can wait longer to sample the current after a potential modulation, thereby allowing the capacitative current to decline, enhancing the proportion of faradaic current. Although nonfaradaic signal can be minimized instrumentally, it cannot be eliminated entirely. An artificial function is typically fit to the nonfaradaic signal and then subtracted. A quality Hg/Au sensor will have a small enough capacitative current relative to the faradaic signal to allow for this subtraction.

The third criterion for Hg/Au sensor quality is whether the faradaic current response of the electrode associated with electron transfer induced by oxidation or reduction of aqueous constituents is within a normal range. Using Mn(II) to evaluate this third criterion also helps check the first criterion because its reduction occurs near the polarization limit of Hg.

Unless polarized at a reducing potential, Hg-based electrodes will oxidize and lose sensitivity after approximately ten hours [27], although this time can vary depending on the sensor material and size [12,21]. Regardless, the time between electrode calibration and quantitative measurements is finite. Analytical sensitivities need to be adjusted if measurements occur beyond this point. Several electrodes are most likely needed to measure redox constituents at multiple locations *in situ*[16], posing an additional burden of knowing and adjusting several constituents’ calibrations on each electrode.

To determine species concentration, an appropriate voltammetric figure of merit, such as a baseline-subtracted wave height, peak current, or electrical charge (peak area) are measured in standard solutions, and the resulting analytical sensitivity (in units of amperes or coulombs divided by concentration) is used to convert subsequent measurements into concentrations. For the remainder of this discussion, the figure of merit will simply be referred to as current or *i*. Rather than measuring a calibration curve for each analyte and electrode, the pilot ion method calculates the concentration of a constituent measured with an uncalibrated electrode as follows [28]:

(1) $$\frac{i_{\text{pilot}}}{i_{\text{u}}}=K\frac{c_{\text{pilot}}}{c_{\text{u}}}$$

where *i*_pilot_ and *c*_pilot_ are the current and concentration of the pilot ion, *i*_u_ and *c*_u_ are the current and concentration of the unknown constituent of interest, and *K* is the ratio of the calibration slope of the pilot ion (*s*_pilot_) divided by the slope of the constituent of interest (*s*_u_):

(2) $$K=\frac{s_{\text{pilot}}}{s_{\text{u}}}$$

*K* defines the difference in electrode response between the pilot ion and any other constituent, as long as the currents are diffusion-limited (no kinetic or catalytic effects) and directly proportional to concentration. If so, this difference in electrode response should be independent of electrode characteristics, temperature, and solution composition [28]. Incidentally, the term pilot *ion* is used for simplicity and does not mean the constituent needs to be ionic. The meaning of ‘diffusion-limited’ will become clearer when experimental results are described, and the interested reader is referred to chapters 5 and 7 in Bard and Faulkner [24] for a full explanation. Rearranging equation 1, the concentration of the unknown constituent is given by

(3) $$c_{\text{u}}=K\;i_{\text{u}}\left(\frac{c_{\text{pilot}}}{i_{\text{pilot}}}\right)$$

A typical use of the pilot ion method is described in the Additional file 1.

Explicit means by which voltammetric signals can be quantified using the pilot ion method have not been provided in the literature. In several publications [11,13,16,29-33], graphical representations of how O_2_, Fe(II), Mn(II), and S(−II) voltammetric signals are distinguished from background are not shown or explained. And for the pilot ion method, these works reference Meites [16] or Brendel and Luther [11], which in turn cites Meites [28]. The only example provided by Meites [16] to substantiate the pilot ion method involved metal analysis at environmentally exaggerated concentrations using a mercury drop (not solid-state) electrode.

In the present study, we show in detail how to quantify the voltammetric signals of O_2_, Fe(II), Mn(II), and S(−II) and evaluate the accuracy of the pilot ion method. By comparing nine replicate Fe(II) and Mn(II) and twelve replicate S(−II) calibration curves on each of three electrodes, the accuracy of calibrations using the pilot ion method are provided for the analysis of seawater with and without naturally derived dissolved organic carbon (DOC) at two DOC concentrations. The approach is then demonstrated with multi-constituent aqueous solutions and sediment pore waters. In addition to providing a reproducible protocol, this paper is the first to evaluate the accuracy of the pilot ion method under realistic environmental conditions.

## Materials and methods

### Materials

Artificial seawater [34] was made with reagent-grade chemicals and deionized water and stored at 4°C. Na_2_S stock solutions were prepared by dissolving rinsed Na_2_S·9H_2_O crystals in deoxygenated water, followed by iodimetric standardization. Water was deoxygenated by boiling, purging with ultrahigh-purity (UHP) N_2_ and storing in a 98% N_2_, 2% H_2_ anaerobic chamber (Coy Labs; industrial grade chamber gases were deoxygenated using a Pd catalyst). Fe(ClO_4_)_2_·6H_2_O (Alfa Aesar; desiccated under N_2_) was dissolved in deoxygenated deionized water. To remove any Fe(III) originating from the dry reagent, the iron stock solution was filtered once daily three times through a 0.02 μm Anotop membrane. Iron stock solutions (pH 2.8) were stored in the glove box and were re-filtered and colorimetrically standardized [35] on the day of each experiment to remove Fe(III) that inevitably forms despite the low pH of the solution and trace (few ppm) levels of oxygen that can persist inside the anaerobic chamber. Stock solutions made with newly purchased ferrous chloride were found to contain much higher levels of Fe(III) contaminant compared to Fe(ClO_4_)_2_·6H_2_O. While others favor using Mohr’s salt to prepare stock solutions [36], Fe(ClO_4_)_2_·6H_2_O is also a reasonable choice [37] and one that we found lost only 3% Fe(II) or less over 1 d presumably due to filtration of oxidized precipitates. MnSO_4_ was weighed on an analytical balance, dissolved in deionized water, and stored at 4°C. Pony Lake fulvic acid (PLfa) was purchased from the International Humic Substances Society. Fulvic acid from Pacific Ocean water collected from 100 m depth, 170 km southwest of Honolulu, Hawaii was isolated as described by Aiken et al. [38]. Both aquatic organic isolates were weighed using a microbalance in a desiccated chamber, dissolved in artificial seawater, and stored at 4°C. The Pacific Ocean isolate was dissolved to 0.850 (mg C)/L, which is within the medium-to-high range of organic C in the ocean [39], whereas PLfa, derived mostly from algal biomass [40], was dissolved at 26.3 (mg C)/L to resemble conditions in biologically productive estuarine sediment.

### Electrode fabrication

Electrodes were fabricated by a procedure modified from Brendel and Luther [11]. 100 μm Au wire (Surepure Chemetals) was soldered to shielded copper cable and sealed with Epo-tek® 360 in a glass tube with a tip pulled from 5 mm to approximately 0.8 mm diameter. The tip was flattened by sanding with 400-grit and successively polished by hand using Buehler Metadi 15, 6, 1, and 0.25 μm diamond polishing compounds suspended in AB Metadi lubricant on Buehler TexMet® (15 and 6 μm) and MicroCloth® (1 and 0.25 μm) affixed to a rotating pedestal mounted on a DC motor equipped with an HY152A AC/DC converter. All electrodes were inspected with a 100x microscope throughout the polishing process, verifying a mirror finish before electroplating with Hg. Without delay, the polished electrode tip was rinsed and placed in a 0.1 M Hg(NO_3_)_2_/0.05 M HNO_3_ solution deaerated with UHP N_2_. Hg was electroplated onto the Au disc at −0.1 V vs. Ag/AgCl/[saturated KCl] for 4 min. The plated electrode was then polarized at −9 V in 1 M NaOH for 90 s [41], rinsed with water and 0.01 M HClO_4_ to remove hydroxide, and stored overnight in deionized water. Unless stated otherwise, electrodes were (re)plated, polarized, and stored in deionized water one day prior to performing calibration measurements.

### Voltammetric analysis of model seawaters

All analyses were performed at 21 ± 2°C. An Autolab PGSTAT12 potentiostat was used for all voltammetric measurements (Metrohm-Autolab B.V. (formerly Eco Chemie), Utrecht, The Netherlands). By convention, reduction currents are negative while oxidation currents are positive, which is the opposite of some systems [23]. Data acquisition, first-derivative calculations, linear and 4^th^-order polynomial fitting, baseline subtractions, and peak analyses were performed with Nova versions 1.6 and 1.7 (Metrohm-Autolab). Non-linear baseline functions were fit to the background signal by choosing three data points on the volammogram on the electropostive side of the region of interest and two points on the negative side. A 4^th^-order polynomial that intersects those five points is then fit. Regression of calibration data, peak deconvolution, statistical analyses (analysis of variance (ANOVA) and chi-squared calculations), and figure data plotting were performed with Igor Pro (Wavemetrics, Portland, Oregon, USA). The electrochemical cell consisted of a 100 μm Hg/Au amalgam working electrode, Ag/AgCl/[saturated KCl] reference electrode, and Pt auxiliary electrode placed in a three-port, 250 mL glass flask that resided inside a polyethylene glove bag (Glas-Col) mounted within an electrically grounded copper screen (Faraday cage) to shield the system from electrical noise. The glove bag was left open to the air to perform cyclic voltammetric analysis of aerated seawater. The system was then deaerated by purging the glove bag under positive pressure with UHP N_2_ for at least 1 h. Unless stated otherwise, S(−II), Fe(II), and Mn(II) stock solutions were added individually to the flask inside the glove bag under continuous UHP N_2_ flow under positive pressure. The pH of artificial seawater was 8.2 ± 0.1 throughout MnSO_4_ addition. To slow Fe(II) oxidation, seawater pH was adjusted prior to and maintained during the Fe(ClO_4_)_2_ additions at 6.0 ± 0.5 with CO_2_ and N_2_ flow through the glove bag. Acidification is permissible because pH does not affect the electron transfer kinetics from the electrode to Fe(II) [10,11]. However, the solution should not be acidified too much, because a H^+^ reduction signal may obscure any Fe(II) signal [10]. Solutions were analyzed after each standard addition with three Hg/Au electrodes in series using a multiplexer (two electrodes were disconnected while the third was used to measure).

S(−II) was measured using normal pulse voltammetry, where the potential (*E*) was scanned from −0.8 to −0.4 V in 0.5 s, 0.005 V steps, with a −0.8 V base potential and 0.05 s pulse time (for further explanation, see Turner et al. [42] and section 7.3.2 in Bard and Faulkner [24]). O_2_ was measured with cyclic voltammetry (five scans from −0.1 to −1.7 V and back at 0.1 V/s in −7 mV steps, 0.07 s interval time). Fe(II) and Mn(II) were measured by three techniques: first, cyclic voltammetry (five scans from −0.1 to −1.7 V and back at 0.1 V/s in −7 mV steps), then linear sweep voltammetry (−0.8 to −1.7 V at −0.1 V/s in −7 mV steps), and finally square-wave voltammetry from −0.8 to −1.7 V in −5 mV steps and 25 mV amplitude pulses at 8 Hz frequency (0.04 V/s; 0.125 s interval time). The electrode was held at −0.4 V for 10 s prior to cyclic voltammetry or −0.8 V for 10 s prior to linear sweep and square-wave voltammetry. The uppermost potential (−0.1 V) was chosen to prevent oxidation of the Hg/Au sensor. We chose a scan rate of 0.1 V/s and a step potential of 7 mV for cyclic and linear sweep scans and 8 Hz for square-wave scans to ensure that the measurement interval time was long enough for the potentiostat to have sufficient bandwidth (100 Hz) in the low current range to accurately measure the 1 to 10 nA currents typically sensed by the 100 μm-diameter Hg/Au electrodes.

### Voltammetric analysis of sediment

To test the electrodes in natural sediment and evaluate data processing approaches with signals measured in complex media, several sediment manipulations were performed in the laboratory. Surface sediments (top 0–2 cm) from a salt pond in South San Francisco Bay, CA (SPA8S3) and a coastal marsh in Mississippi (MSM2) were collected and stored in completely filled mason jars, chilled on wet ice, brought to the laboratory for further sub-sampling under controlled anoxic conditions [43], and refrigerated at 4°C to retard microbial processes and prevent sample degradation until use. MSM2 sediment contained 57% solids, consisting of 70% silt and clay (<64 μm), 5.6% organic matter (LOI), and 15 μmol chromium-reducible sulfur (CRS) per gram of wet sediment. SPA8S3 sediment contained 47% solids, of which 70% were silts and clays, 13% organic matter, 61 μmol/g CRS, and 70 mg/L dissolved organic carbon in the pore water. Sediments were mixed with deaerated seawater in a three-port, 250 mL glass flask. The electrode tip was placed within the pore space of the sediment. A set of voltammetric scans was performed in the anoxic pore water. The sediment was then oxygenated by injecting aerated seawater followed by periodic measurements.

## Results and discussion

### Preliminary electrode evaluation

**Figure 1:**
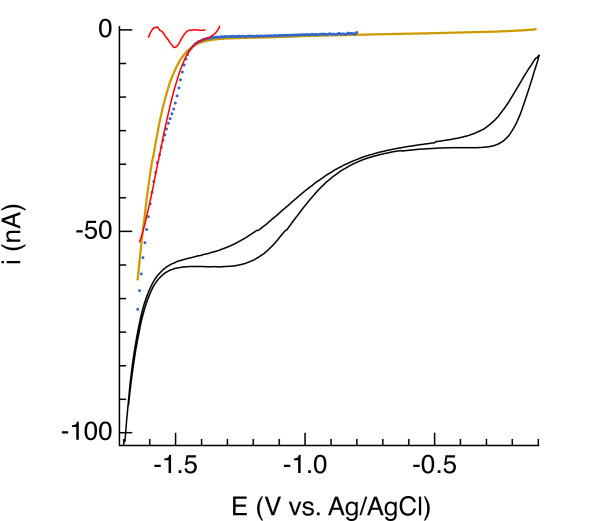
** The black line is a cyclic voltammogram showing current (*i*, in nanoamperes) versus potential (*E*, in volts) during reduction of O**_**2**_**to H**_**2**_**O**_**2**_**and H**_**2**_**O**_**2**_**to H**_**2**_**O in artificial seawater equilibrated with air at 20°C.** The brown line is a linear sweep voltammogram (LSV) of deaerated seawater. The dotted blue line is an LSV of deaerated seawater containing 110 μM Mn(II), fit by a 4^th^-order polynomial to yield a peak representing the faradaic Mn(II) current shown in red.

In aerated seawater, all Hg/Au electrodes used to perform calibration and pilot ion measurements yielded well-defined O_2_ and H_2_O_2_ reduction waves and Na^+^ reduction more negative than −1.6 V, indicating that the tips of the Au wires were well coated with Hg/Au amalgam. An example is shown by the black curve in Figure 1. As the potential becomes negative enough to reduce O_2_, the electrode-water interface becomes depleted of O_2_. This depletion creates a concentration gradient that drives O_2_ diffusion toward the interface such that the current is eventually limited by the rate at which O_2_ diffuses. This mass transport-limited current [24] evolves into the second wave where H_2_O_2_ is reduced to H_2_O, followed by partial Na^+^ reduction and reversal of the potential sweep. In deaerated seawater (brown line in Figure 1), nonfaradaic current was minor compared to faradaic signals arising from electron transfer to O_2_ and H_2_O_2_ in aerated solution. The linear sweep voltammetric signal measured in seawater amended with 110 μM MnSO_4_, shown with a blue dotted line in Figure 1, yields a peak current (after background removal shown in red) equivalent to a sensitivity of 0.043 nA/μM, which we will see is typical and similar to what has been previously reported [11]. On the basis of these data, we concluded that this electrode met our criteria (see introduction) and therefore was fit for use. All other results presented in this paper were obtained using electrodes that were similarly determined to be fit for use.

**Figure 2:**
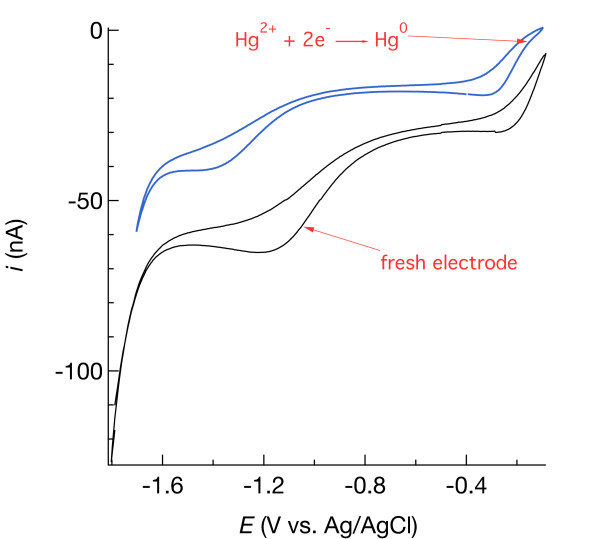
Cyclic voltammograms in aerated artificial seawater measured with a freshly plated Hg/Au amalgam electrode (black curve) and with the same electrode after storage in water for four days in open circuit (blue curve).

Hg/Au amalgam oxidizes over a period of days when an electrode is left in open circuit (i.e., no potential applied). An example of how a cyclic voltammetric signal in aerated water changes as a result of Hg/Au oxidation is shown in Figure 2. After four days, the overall signal is diminished, as is the separation between O_2_ and H_2_O_2_ currents used to quantify dissolved O_2_. In addition, the presence of oxidized mercury (Hg^+^ or Hg^2+^) is revealed by an inflection at −0.15 V (blue curve in Figure 2), which the scan measured with the fresh electrode does not have (black curve in Figure 2). The partially oxidized sensor can still be used, but detection limits will likely be worse. In the present example, the air-saturated, baseline-corrected O_2_ current decreased from 34.1 nA to 16.9 nA after four days, a 50% decline in sensitivity. While evaluating the utility and accuracy of the pilot ion method, electrodes used in this study were either replated the day before they were used to obtain calibration data, or they were continually polarized. In the latter case, one electrode was connected to the potentiostat through a multiplexer and polarized at −0.8 V for 10 min. while the other electrodes were connected to the multiplexer but disconnected from the potentiostat. After 10 min., the multiplexer disconnected the first electrode and connected the second electrode to the potentiostat for 10 min. at −0.8 V, and so on, repeatedly cycling through all electrodes, automated in a procedure programmed using the Nova software. The variability of one electrode on different days was either a consequence of how the electrode was handled (polarized versus replated) or how precise the plating was.

### Quantification of O_2_, S(−II), Fe(II), and mn(II) in model seawater solutions and sediments

**Figure 3:**
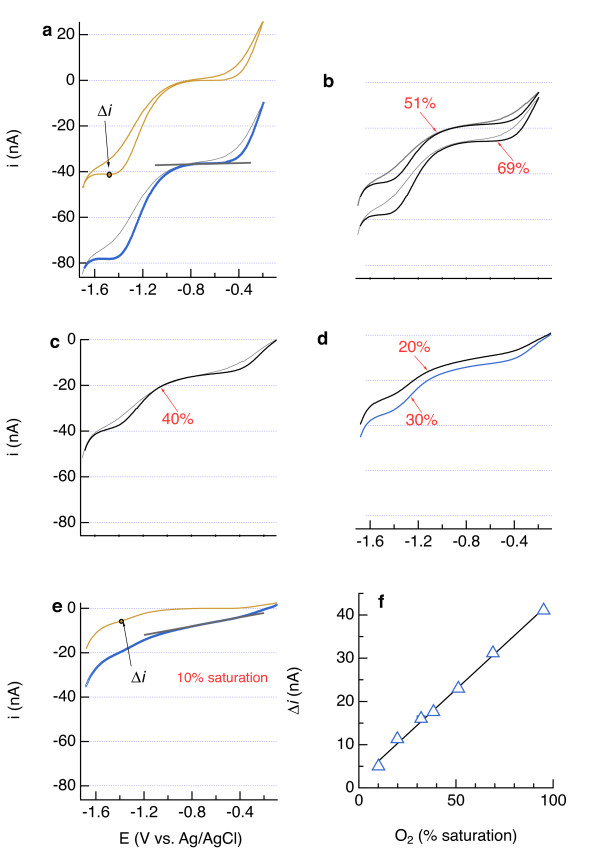
** Cyclic voltammograms during reduction of O**_**2**_**to H**_**2**_**O**_**2**_**and H**_**2**_**O**_**2**_**to H**_**2**_**O in artificial seawater at different degrees of oxygen saturation.** In **a** and **e**, baselines are shown in black and background-subtracted data in brown, from which the inflection point after H_2_O_2_ reduction was plotted vs. O_2_ concentration independently measured with an amperometric membrane electrode (**f**). O_2_ concentration was adjusted by varying the proportions of air and N_2_ blanketing the flask.

Dissolved O_2_ was quantified from the inflection point following the H_2_O_2_ wave after subtracting a linear baseline to the plateau following O_2_ reduction (Figure 3a-e). Such wave heights significantly correlate to O_2_ concentrations independently determined using an amperometric membrane electrode (Figure 3f), as has been found with an optical sensor and Winkler titrations [44]. In practice, voltammetric electrodes are usually calibrated for O_2_ on the basis of a single wave height obtained from an air-saturated solution [44]. A similar approach is also reliable for calibrating amperometric electrodes [45].

During analysis of S(−II) using normal pulse voltammetry, the electrode potential was modulated in cycles. In each cycle, the potential is first held at −0.8 V for 0.5 s, during which time S(−II) does not react, and then the potential is pulsed by +25 mV for 0.05 s before being returned to −0.8 V. With each subsequent cycle, the crest of the pulse increases by 0.005 V (see Figure 4.3.5 in Bard and Faulkner [24] for an illustration of the potential waveform), eventually reaching potentials where S(−II) oxidizes some of the Hg/Au amalgam:

(4) $${\text{Hg}}^{0}+{\text{ SH}}^{-}\to {\text{HgS}}_{\left(\text{s}\right)}+{\text{ H}}^{+}+{\text{ 2 e}}^{–}$$

**Figure 4:**
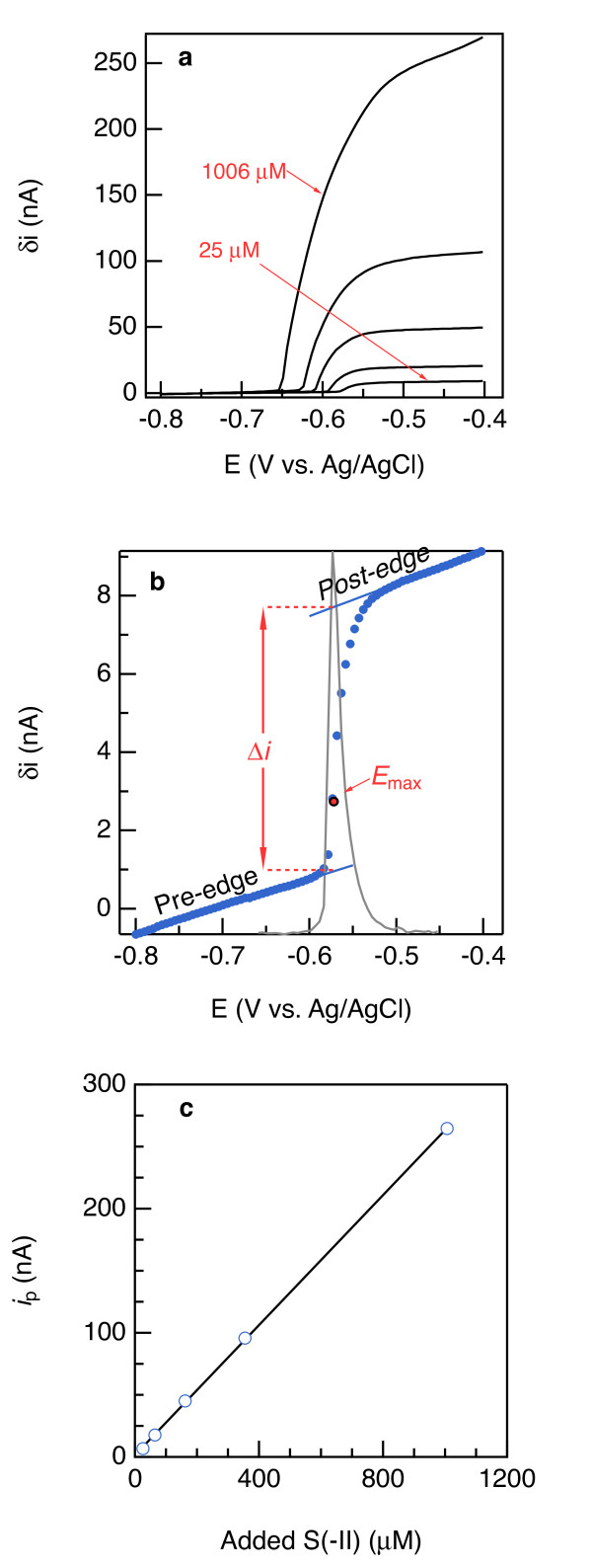
** (a) Current (i) versus potential (E) relationships to quantify S(-II) with normal pulse voltammetry.** (**b**) The dots represent the pulse current δ, in nanoamperes, measured in artificial seawater containing 25.4 μM S(-II). The solid line is its first derivative, the maximum of which locates Emax at which to calculate the current Δi on the basis of the coefficients of the pre- and post-edge regressions before and after the jump in i upon Hg oxidation by S(-II), which is then plotted against added S(-II) concentration to calibrate the electrode (c; see Table 1 for calibration slopes).

**Figure 5:**
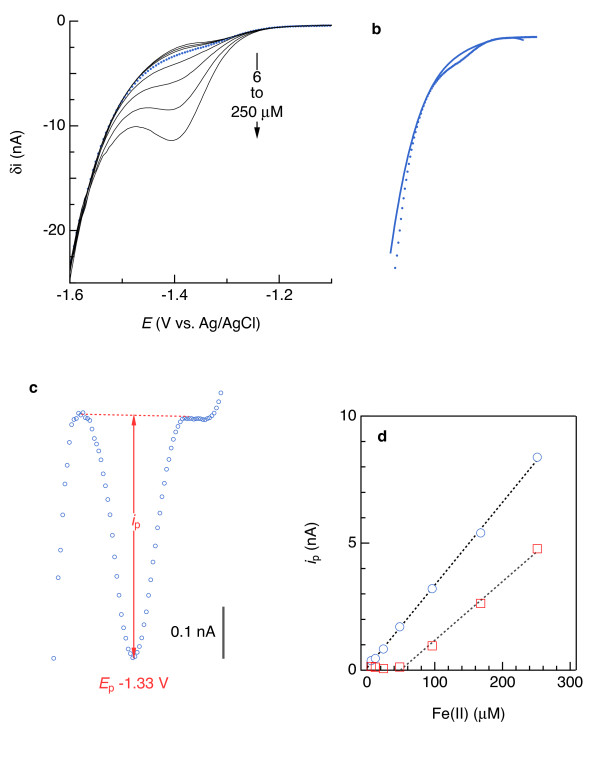
** (a) Current (*i*) versus potential (*E*) relationships for Fe(II) in artificial seawater containing 0.85 (mg C)/L as Pacific Ocean fulvic acid in square-wave mode, where δ*i*is difference between the forward pulse current*i*at*E*– 25 mV and reverse pulse current*i*at*E*** **+ 25 mV.** (**b**) 4^th^-order polynomial baseline fit (solid line; δ*i* = −1.77631·10^-6^ – 5.65237·10^-6^*E* – 6.75637^-6^*E*^2^ – 3.59825·10^-6^*E*^3^ – 7.21311 ·10^-7^*E*^4^) to 12 μM Fe(II). (**c**) Plot of peak current (*i*_p_) due to Fe(II) reduction obtained by baseline subtraction. (d) Calibration curves: blue symbols represent data obtained by 4^th^-order polynomial subtraction, *i*_p_ = 0.0326(9)·[Fe(II)] + 0.1(1), with numbers in parentheses indicating the 90% confidence interval in the last digit and square brackets denoting concentration in μM; R^2^ = 0.9996; red symbols represent data obtained from linear baseline subtractions.

The current generated by this reaction and measured near the end of each pulse (Figure 5a) is well correlated to S(−II) concentrations ranging from 25 to 1,000 μM (Figure 5c). As shown in Figure 5b, this current is determined by linear regression of regions before and after the jump in current and taking the difference between the two functions at the inflection of the jump. Inflections were precisely determined by taking the first-derivative of the voltammogram and choosing the peak of the derivative signal. After the pulse is complete and the electrode potential returns to −0.8 V, any HgS_(s)_ is reductively decomposed, preventing over-accumulation of HgS_(s)_. In tidal mud flats, marshes, and biostimulated subsurface sediment, S(−II) concentrations can reach 1 mM or more [46]. Under these conditions, excessive formation of HgS_(s)_ on the sensor must be avoided to properly quantify the S(−II) that is converted to HgS_(s)_[47,48].

**Figure 6:**
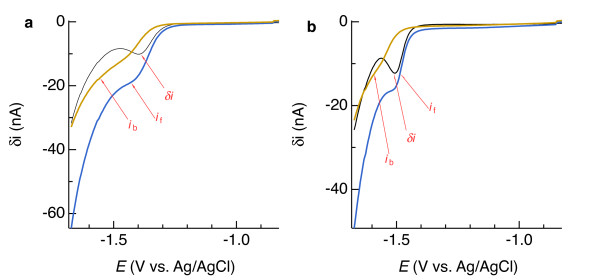
Breakdown of square-wave voltammetric signal (δi, black curves) in to forward (blue) and reverse (brown) pulse currents measured in artificial seawater containing (a) 250 μM Fe(II) or (b) 110 μM Mn(II).

**Figure 7:**
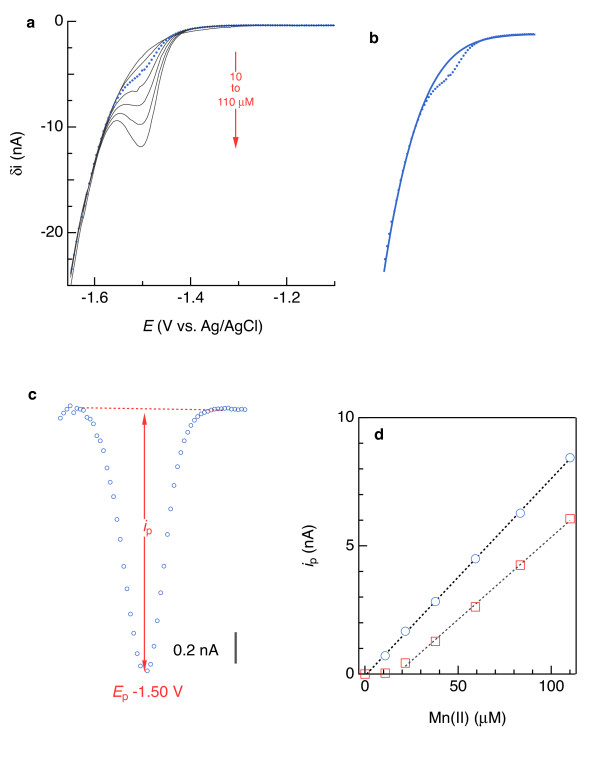
**(a) Current (*i*) versus potential (*E*) relationship for Mn(II) in artificial seawater containing 0.85 (mg C)/L as Pacific Ocean fulvic acid in square-wave mode, where δ*i*is equivalent to*i*measured at the end of the*E*– 25 mV pulse minus*i*measured at*E*** **+ 25 mV pulse.** (**b**) 4^th^-order polynomial baseline fit (solid line; δ*i* = −7.22469·10^-6^ – 2.14916·10^-5^*E* – 2.39998^-5^*E*^2^– 1.19248·10^-5^*E*^3^ – 2.22469 ·10^-6^*E*^4^) to 21.6 μM Mn(II). (**c**) Plot of peak current (*i*_p_) due to Mn(II) reduction obtained by baseline subtraction. (**d**) Calibration curves: blue symbols represent data obtained by 4^th^-order polynomial subtraction, *i*_p_ = 0.077(1)·[Mn(II)] – 0.1(1), with figures in parentheses indicating the 90% confidence interval in the last digit and square brackets denoting concentration in μM; R^2^ = 0.9998; red symbols represent data obtained from linear baseline subtractions

Reduction of Fe(II) to Fe(0) at a Hg/Au electrode detected Fe(II) as low as 6 μM (Figure 6). Fe(II) reduction typically occurred at approximately −1.34 V and the width of the reduction wave ranged from 50 to 120 mV, partially overlapping Na^+^ reduction signal. Fourth-order polynomial baselines (Figure 6b) yield nearly symmetrical peaks from which to determine the peak height (*i*_p_) used to quantify Fe(II). Eighteen calibration curves ranging from 6–300 μM had linear coefficients of determination (R^2^) > 0.999 (Figure 6c,d), of which 6 had offsets (y-axis intercepts) that were statistically indistinguishable from zero at the 90% confidence level. The lowest offset (−0.56 nA) corresponds to a concentration axis intercept of 10 μM, whereas the highest offset (0.15 nA) is approximately half of the peak current measured in the solution containing 6 μM Fe(II), suggesting a 10 μM Fe(II) quantification limit. For all three electrodes and three aqueous media (seawater with and without DOC), electrode sensitivities for Fe(II) with linear sweep voltammetry (LSV) data did not appreciably differ from square-wave voltammetry (SWV): *s*_LSV_/*s*_SWV_ ranged from 0.93 to 1.15. This similarity is due to the kinetic limitations of the Fe(II)/Fe(0) redox couple at the electrode interface. The cathodic (negatively oriented) forward pulse of the square-wave cycle reduces Fe^2+^ to Fe^0^, but the anodic (positively oriented) reverse pulse does not appreciably oxidize Fe^0^ and so generates minimal anodic current that would otherwise enhance δ*i*, which is equal to *i*_f_ – *i*_r_ (Figure 7a) [24]. However, the anodic pulse current includes some of the nonfaradaic (background) signal that, when subtracted from *i*_f_, leaves a more discernible Fe(II) signal in the δ*i* vs. *E* curve (compare blue and black curves in Figure 7a). As explained later, the remaining background signal needs to be removed with a mathematical function.

Reduction of Mn(II) to Mn(0) at a Hg/Au electrode also yields quantifiable peaks upon subtraction of 4^th^-order polynomial background signals (Figure 4). Peak potentials (*E*_p_) were about −1.5 V, and so the Mn(II) portion of the curve overlaps more with the Na^+^ signal than does Fe(II). This degree of overlap, which we observed with all of the electrodes used in this study, did not preclude Mn(II) detection down to about 10 μM, providing further evidence that the tips of the sensors’ Au wires were well-coated with Hg/Au amalgam as described in the previous section. As with Fe(II), SWV yields a more easily discernible analytical signal for Mn(II) compared to LSV (compare blue and black curves in Figure 7b). Mn(II) sensitivities were, on average, more than twice those of Fe(II) (Tables 1 and 2).

**Table 1:** Hg/Au electrode sensitivities (calibration slopes) measured in artificial seawater

	**Sensitivity (10**^**-2**^ **nA/μM)**		
	**Mn(II)**	**Fe(II)**	**S(-II)**
*Artificial seawater (no organic carbon)*			
E1	7.4(1), 5.6(1)	2.71(7)	31.6(3), 30.0(3)
E2	7.7(1), 6.5(1)	3.11(8)	31(1), 23.1(2)
E3	6.9(1), 12.5(3)	5.3(1)	24.7(5), 55.3(4)
Artificial seawater with 0.85 (mg C)/L Pacific Ocean fulvic acid			
E1	7.7(2)	3.26(9)	26.2(4)
E2	6.3(2)	2.11(6)	17(1)
E3	6.9(2)	2.66(8)	21.9(8)
Artificial seawater with 26 (mg C)/L Pony Lake fulvic acid			
E1	-	5.4(1)	46(2)
E2	-	2.44(5)	21.59(5)
E3	-	2.77(6)	25.0(3)

Figures in parentheses indicate the 90% confidence interval in the last digit.

**Table 2:** Comparison of calibration slope ratios for Mn(II) and Fe(II) for different electrodes

**Date**	**Electrode**	**s**_**Mn**_	**s**_**Fe**_	***K*** = **s**_**Mn**_**/s**_**Fe**_	**s**_**S(-II)**_	***K*** = **s**_**Mn**_**/s**_**S(-II)**_	***i*(nA)**	**s**_**Oxygen**_	***K*** = **s**_**Mn**_**/s**_**Oxygen**_
8/19/10	1	0.05607	0.0271	2.07	0.300	0.187	29.3	0.211	0.266
	2	0.06931	0.0311	2.23	0.231	0.300	31.6	0.227	0.305
	3	0.1246	0.0537	2.32	0.553	0.225	45	0.324	0.385
8/20/10	1	0.077	0.0326	2.36	0.262	0.294	27.8	0.200	0.385
	2	0.0633	0.0211	3.00	0.168	0.377	20.2	0.145	0.436
	3	0.06991	0.0266	2.63	0.219	0.319	23.7	0.171	0.410
average (all *K* values)				2.43		0.284			0.364
standard deviation				0.33		0.068			0.065
95% confidence interval				0.27		0.054			0.052
C.I./average				11%		19%			14%

**Figure 8:**
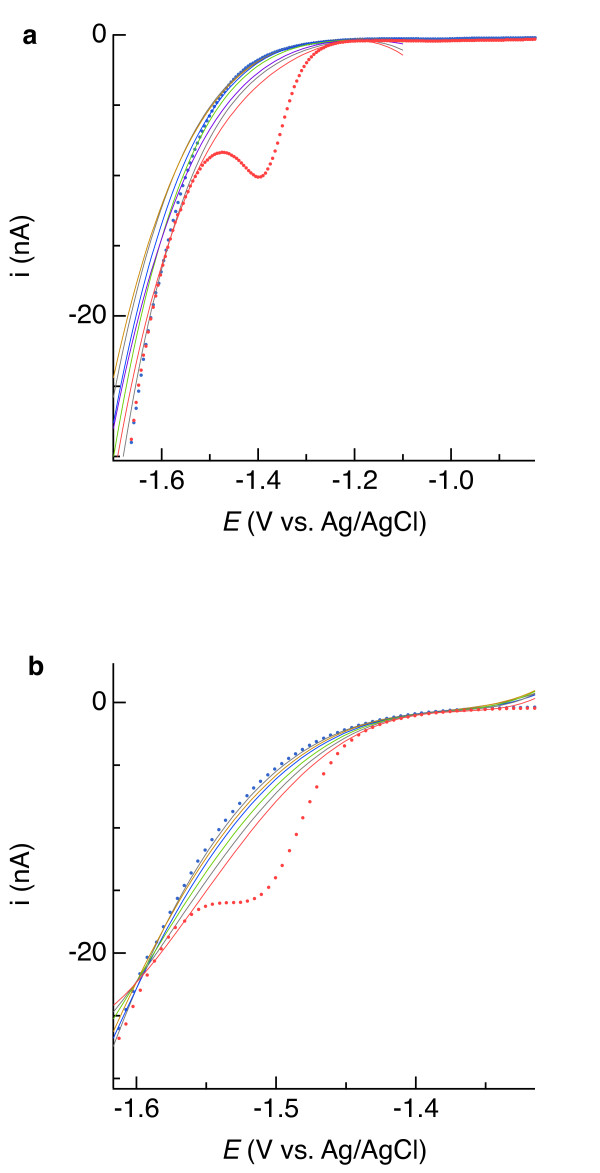
** Analyte-free square-wave voltammograms (SWV; dotted blue lines) with 4**^**th**^**-order polynomial baselines fitted to Fe(II) (a) and Mn(II) (b) calibration data (solid lines).** The red dotted lines are the SWVs for 253 μM Fe(II) and 110 μM Mn(II).

**Figure 9:**
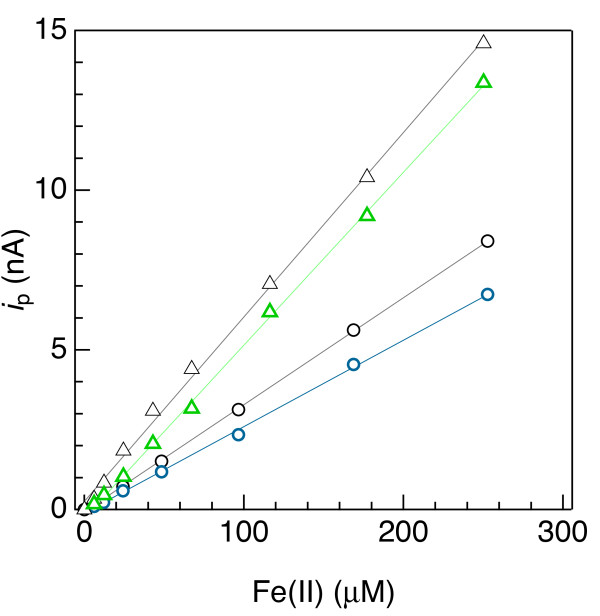
** Calibration curves obtained by subtracting individually fitted 4**^**th**^**-order baseline functions (blue and green symbols) and by subtracting one common Fe(II)-free (blank) square-wave voltammogram (SWV) from each Fe(II)-bearing SWV (black symbols).** The circles represent measurements in artificial seawater, and the triangles represent analyses of artificial seawater amended with 26 mg C/L as Pony Lake fulvic acid.

Since baseline subtraction for voltammetric Fe(II) and Mn(II) analysis has rarely been mentioned [23], it may be unclear if a non-linear baseline is appropriate or necessary. As shown by the blue dotted lines in Figure 8a and b, as the voltammetric scan proceeds towards more negative potentials, the current-potential relationship without Fe(II) or Mn(II) is a relatively flat line followed by a sharp downward curve resulting from Na^+^ reduction. For comparison, the voltammetric signals for 253 μM Fe(II) and 110 μM Mn(II) are shown with dotted red lines. Non-linear, 4th-order polynomial baselines fitted to the Fe(II)- or Mn(II)-bearing data follow the same topology as the Fe(II)- and Mn(II)-free curves, indicating that they are appropriate representations of the background currents we wish to remove: nonfaradaic current plus faradaic current caused by Na^+^ reduction. As Fe(II) or Mn(II) concentration increases, the background signal shifts toward more negative currents (Figure 8). This phenomenon affects calibrations. For example, if the Fe(II)-free voltammogram shown by the blue dotted curve in Figure 8 is subtracted from each Fe(II)-amended seawater voltammogram, the resulting calibration slope is significantly higher (0.0335(3) nA/μM, with the 90% confidence interval of the last digit in parentheses) than the slope based on individual baseline subtractions (0.0271(7) nA/μM), as shown in Figure 9. For the synthetic seawater analyses represented in Figure 8, subtracting blank voltammograms degraded the quality of Fe(II) and Mn(II) calibration curve regressions, in that the curves based on blank-subtracted data have significant non-zero offsets, whereas fitting individual baselines yields offsets that are statistically indistinguishable from zero at 90% confidence.

Analyses of Fe(II)- or Mn(II)-bearing seawater indicate that subtracting a linear baseline to obtain *i*_p_ for Fe(II) or Mn(II) is error-prone and of limited use, while 4^th^-order polynomial subtractions provide analytical sensitivity similar to what is determined in simpler, one-component systems. Results, of which examples are provided by the red symbols in Figure 6d and 4d, consistently indicated that linear baselines produced appreciably offset Fe(II) and Mn(II) calibration curves and precluded detection of less than approximately 50 μM Fe(II) and 20 μM Mn(II). By contrast, Fe(II) and Mn(II) signals as low as 6 μM were detectable, and 10 μM quantifiable, upon subtraction of 4^th^-order polynomials, as shown by the blue symbols in Figure 6d and 4d. These results suggest that linear baselines may be useable for solutions containing at least 20 to 50 μM Mn(II) or Fe(II), but the extra effort to fit a 4^th^-order polynomial may be justified to quantify Fe(II) and Mn(II) below those concentrations.

**Figure 10:**
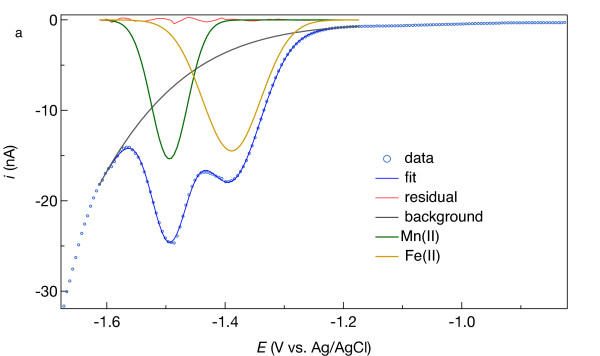
Deconvolution of a square wave voltammogram of artificial seawater containing 548 μM Fe(II) and 110 μM Mn(II).

**Figure 11:**
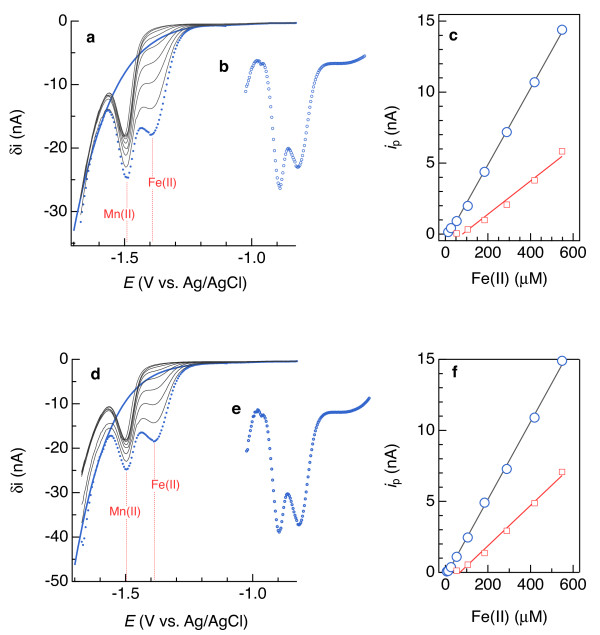
** Calibration analysis of Fe(II) in artificial seawater that also contained 110 μM Mn(II):** (**a**) square wave voltammograms and example baseline subtraction (solid blue line) of dotted blue data; (**b**) baseline-subtracted voltammogram, and (**c**) calibration curves obtained by 4^th^-order polynomial (blue) and linear (red) baseline subtractions. Panels d-f provide the same results as a-c for a second electrode.

**Figure 12:**
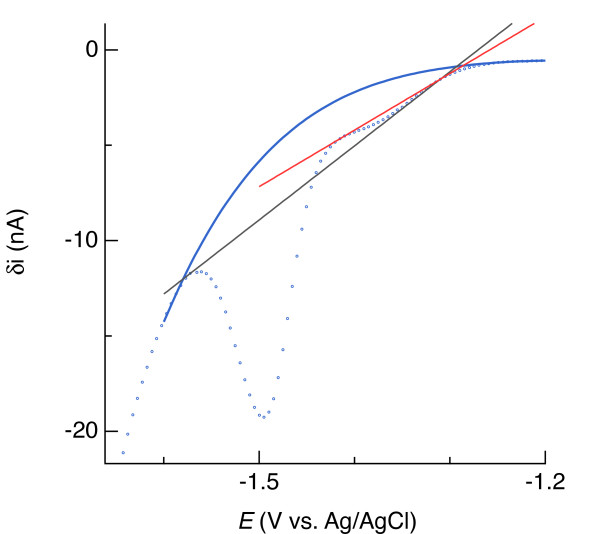
** Baseline fitting of a square-wave voltammogram of 105 μM Fe(II) in artificial seawater (dotted blue curve) that also contained 110 μM Mn(II).** The solid blue line is a 4^th^-order polynomial and the red and grey lines are attempts to fit linear baselines.

Redox constituents like Fe(II) and Mn(II) can co-exist in sediments [16,49]. To evaluate linear and 4^th^-order polynomial subtractions in systems approaching this complexity, we analyzed artificial seawater spiked with 170 μM Mn(II) and a range of Fe(II) concentrations and sediments that contained both Mn(II) and Fe(II). 4^th^-order polynomials facilitated deconvolution of overlapping Fe(II) and Mn(II) signals measured in artificial seawater into symmetric peaks (e.g., Figure 10) down to 13 μM Fe(II). Calibration curves derived from 4^th^-order polynomial subtractions yielded average Fe(II) sensitivities of 0.0268(8) and 0.0272(7) nA/μM for the two examples shown in Figure 11 (c and f, blue symbols), which are similar and even identical, in some cases, to sensitivities similarly determined in the absence of an overlapping Mn(II) signal (Table 1). The regressions of the Fe(II) calibration curves were of similar quality in the presence and absence of Mn(II): χ_ν_^2^ = 0.047 and 0.039 with Mn(II) present, compared to χ_ν_^2^ = 0.010 on average in its absence (χ_ν_^2^ is the reduced chi-squared goodness-of-fit parameter). There are two possible ways of fitting linear baselines to these overlapping signals: across the Fe(II) peak, as shown in red in Figure 12, or across both peaks, shown in grey in Figure 12 and restricted to signals from at least 180 μM Fe(II). Adopting the less restrictive approach, linear regressions of calibration points obtained following linear baseline subtractions were worse than those resulting from 4^th^-order polynomial subtractions (red (χ_ν_^2^ = 0.119 and 0.076) vs. blue (χ_ν_^2^ = 0.047 and 0.039) lines in Figure 11c and f). In artificial seawater, 4^th^-order polynomial subtractions produce similar electrode sensitivities for systems containing Fe(II) or Mn(II) in isolation or a mixture of the two. This consistency is evidence that 4^th^-order polynomials are more appropriate than linear baselines for Fe(II) and Mn(II) quantification.

**Figure 13:**
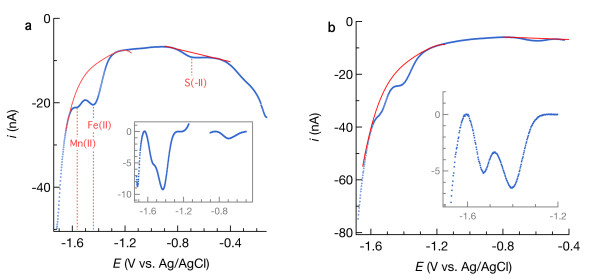
** Square-wave voltammogram of sediment from (a) a Mississippi Gulf Coast marsh and (b) salt pond in South San Francisco Bay.** Solid red lines show the baselines and the subtracted data are inset.

**Figure 14:**
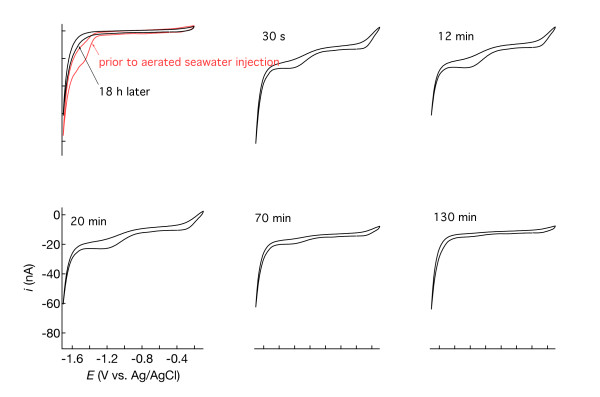
** Cyclic voltammograms measured in Mississippi Gulf Coast marsh sediment after oxygenation by aerated seawater injection.** The time since injection is indicated in each panel.

**Figure 15:**
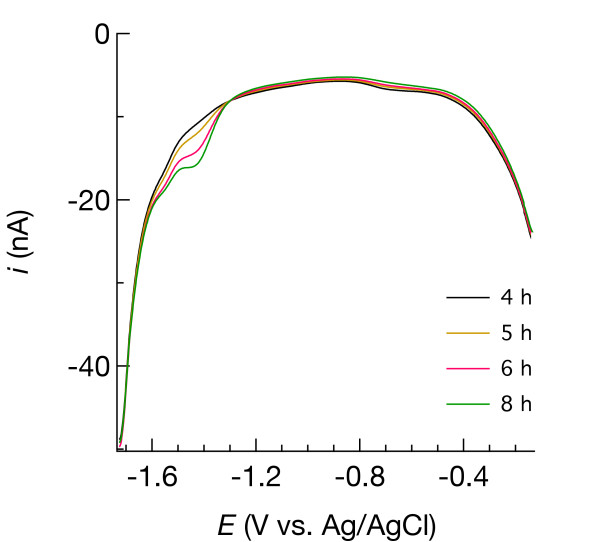
Square-wave voltammograms measured following anaerobiosis of the Mississippi Gulf Coast marsh sediment in a laboratory manipulation experiment.

**Figure 16:**
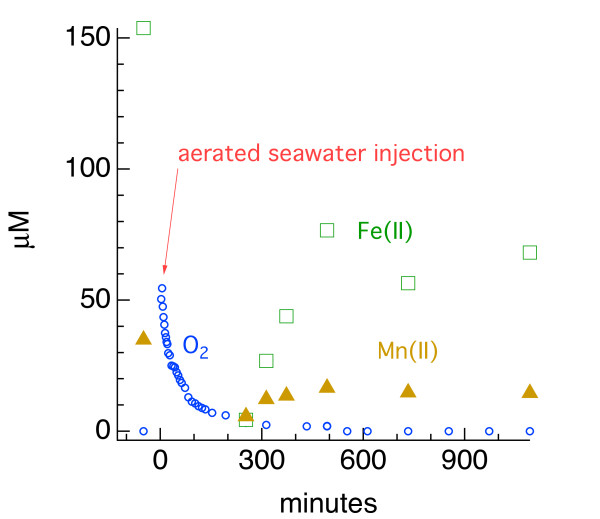
Concentrations of redox constituents obtained from voltammetric analysis of the Mississippi Gulf Coast marsh sediment during the laboratory manipulation experiment.

Laboratory analysis of sediments from two contrasting locations—a Mississippi Gulf Coast marsh and a salt pond in South San Francisco Bay—indicate that voltammetric signals measured with Hg/Au electrodes in sediment pore water resemble those measured in synthetic seawater. Voltammetric analysis revealed the presence of Fe(II), Mn(II), and reduced sulfur in both sediments (Figure 13). The overlapping Fe(II) and Mn(II) signals resemble those in synthetic seawater, before and after 4^th^-order baseline subtraction (Figures 10, 11b and e, and 13 insets). After analyzing the anoxic Mississippi marsh sediment (Figure 13a), the sediment was oxygenated by adding aerated seawater. A sequence of cyclic and square-wave voltammetric analyses were then performed to closely monitor how the systems changed over time; example voltammograms are shown in Figure 14 and 15, yielding the time-resolved redox behavior plotted in Figure 16. These experimental results demonstrate that the baseline fitting approach described can be used to quantify multiple redox constituents in two contrasting sediments across a range of redox conditions that change in time. Other natural systems could contain constituents that result in more complicated voltammetric signals. The user must decide on a case-by-case basis whether data contain useable quantitative information and decide on how to isolate the faradaic signal(s). The utility of linear versus non-linear baselines will depend on the chemical properties of samples and other circumstances that are beyond the scope of this paper. Specific considerations to keep in mind are discussed in the section *Guidelines for baseline fitting*.

### Electrode variability

On three separate occasions, Fe(II) and Mn(II) calibration curves were measured with three electrodes in succession. The following comparisons refer to the analytical sensitivities (calibration slopes) provided in Table 1 and do not denote statistical variability, as three electrodes do not constitute a statistical population. On a given day, sensitivities varied from electrode to electrode (relative to the average) by 13 to 76% for Fe(II) (40% on average with a median of 43%), and 4 to 76% for Mn(II) (24% on average with a median of 11%). On four separate occasions, S(−II) calibration curves were measured with three electrodes in succession. On a given day, sensitivities varied from electrode to electrode by 0.3 to 82% for S(−II) (37% on average with a median of 26%). Since all of these electrodes met the three quality criteria explained earlier, this degree of analytical variability reflects the difficulty of reproducing electrode surfaces by hand.

To restore sensitivity, electrodes were periodically replated with Hg and polarized at −9 V for 90 s. In one experiment, Mn(II) and S(−II) were measured separately with three electrodes at several concentrations in seawater, after which the electrodes were placed in deionized water in open circuit. A day later, these electrodes were replated, polarized, stored overnight in deionized water, and then used again to separately measure Mn(II) and S(−II) in seawater. The sensitivity of each electrode to Mn(II) changed by 10, 27, and 58% from the values measured two days before. The sensitivity of each electrode to S(−II) changed by 5, 31, and 76%. In most cases, the reconditioning procedure did not restore the electrode to its original sensitivity.

If possible, sensitivity can be better maintained by continually polarizing an electrode to prevent oxidation of the Hg/Au sensor. After measuring aerated seawater without added dissolved organic carbon, three electrodes were polarized at −0.8 V ten minutes at a time in series (using a multiplexer) for 16 hours. They were then used to measure cyclic voltammograms in aerated seawater amended with 0.85 mg C/L as Pacific Ocean fulvic acid. For two electrodes, Δ*i* for O_2_ decreased very little: 30.7 to 30.2 nA and 21.3 to 20.2 nA; the Δ*i* for the third electrode dropped more, from 48.3 to 29.0 nA (some small difference can be attributed to the addition of dissolved organic carbon). Two out of three of these results are consistent with an experiment reported by Luther et al. [13], in which Hg/Au electrodes were stable for as long as two months when polarized at potentials sufficiently negative to prevent Hg/Au oxidation. It is important to point out that Luther et al. continuously polarized their electrodes, whereas electrodes were continually polarized 10 minutes out of 30 in this study. Polarizing electrodes even continually may be unfeasible if a potentiostat is used for other purposes or under logistical constraints like traveling to a field site. In addition to correcting electrode-to-electrode variability caused by hand fabrication, the pilot ion method would be useful to account for changes in sensitivity due to reconditioning or oxidation.

### Accuracy of the pilot ion method

To determine O_2_, S(−II), Fe(II), and Mn(II) concentrations with uncalibrated electrodes, we first verified that the current response for each of these constituents is directly proportional to their concentration (Figure 3, Figure 6, Figure 4, and Figure 11). Next, we needed to determine if the quotient of analytical sensitivities between the pilot ion and constituent of interest (*K* in equation 1) is, in fact, independent of the electrode used. Contemporaneously measured calibration slopes for Mn(II) and Fe(II) using square-wave voltammetry indicate that $K\left(s_{\text{Mn}}/s_{\text{Fe}}\right)$ averaged 2.4 ± 0.3 (95% confidence interval); that is, at 95% confidence, the difference in response to Mn(II) and Fe(II) varies by no more than 11% from one Hg/Au electrode to another (Table 2). We conclude on the basis of this result that $K\left(s_{\text{Mn}}/s_{\text{Fe}}\right)$ is independent of the Hg/Au electrode used, and so Mn(II) should be a reasonably accurate pilot ion for Fe(II). Mn(II) is a convenient pilot ion because it does not oxidize appreciably over experimental time scales and therefore is easier to measure.

Using current response data for O_2_ in air-saturated media and Mn(II) calibration slopes, $K\left(s_{\text{Mn}}/s_{{\text{O}}_{2}}\right)$ averaged 0.36 ± 0.05 (95% confidence interval; Table 2); that is, the difference in response to Mn(II) and O_2_ varies by no more than 14% from one Hg/Au electrode to another. Although slightly more variable than $\left(s_{\text{Mn}}/s_{\text{Fe}}\right)$, $K\left(s_{\text{Mn}}/s_{{\text{O}}_{2}}\right)$ is arguably independent of the Hg/Au electrode used, and so Mn(II) should be a good pilot ion for O_2_. However, as mentioned earlier, most researchers calibrate each electrode directly for O_2_ by measuring a single air-saturated current response.

The slope ratio *K* is more variable for Mn(II) and S(−II) measured by square-wave (SW) and normal pulse voltammetry (NPV), respectively: $s_{\text{Mn}}/s_{\text{s}\left(-\text{II}\right)}$ averaged 0.28 ± 0.05 (95% confidence interval; Table 2); that is, at 95% confidence, the difference in electrode response to Mn(II) and S(−II) varies by almost 20% from one Hg/Au electrode to another. The higher variability in *K* for $s_{\text{Mn}}/s_{s\left(-\text{II}\right)}$ reflects differences in Hg/Au oxidation by S(-II) to form HgS and Mn(II) reduction and Mn(0) sorption/amalgamation with Hg/Au. For example, comparing one electrode to another, a 10% difference in the efficiency of Mn(II) reduction to Mn(0) apparently does not imply a 10% difference in the efficiency of Hg/Au oxidation by S(-II). As a result, the *K* values for each electrode can appreciably differ. In summary, depending on the demands of the user, Mn(II) should be a good pilot ion for Fe(II), somewhat less good for O_2_, and perhaps unacceptable for S(-II) quantification.

To check these preliminary conclusions, Fe(II) concentrations calculated using Mn(II) as the pilot ion were compared to their actual values. These data are provided in Table 3 and are available electronically. For concentrations of about 15 μM Fe(II) and higher, the concentrations predicted by the pilot ion method were 0.7 to 24% different from their actual values (13% on average for 35 data). For example, on August 20, 2010, electrode E1 yielded a baseline-corrected peak current $\left(i_{\text{pilot}}\right)$ of 8.44 nA for 110 μM Mn(II) $\left(c_{\text{pilot}}\right)$ The slope ratio $K\left(s_{\text{Mn}}/s_{\text{Fe}}\right)$ is, on average, 2.43 (Table 2). In artificial seawater amended with 48.0 μM Fe(II), a baseline-corrected peak current $\left(i_{\text{u}}\right)$ of 1.71 nA was measured. Using equation 3, we would predict the Fe(II) concentration to have been.

**Table 3:** Comparison of actual Fe(II) concentrations to those predicted by the pilot ion method

***K =*** **2.43**			
**i(Fe), nA**	**predicted*c***_**Fe**_**μM**	**actual*c***_**Fe**_	**Difference**
**August 19, 2010**			
*Electrode 1*			
*c*_pilot_	110		
*i*_pilot_	6.02		
0.0976	4.342	6.042	−28%
0.2265	10.076	12.08	−17%
0.5873	26.13	24.16	8.1%
1.182	52.58	48.31	8.8%
2.347	104.41	96.5	8.2%
4.541	202.0	168.7	20%
6.734	299.6	252.7	19%
*Electrode 2*			
*c*_pilot_	110		
*i*_pilot_	7.441		
0.3043	10.95	*see above*	81%
0.4153	14.95		24%
0.6217	22.38		−7.4%
1.062	38.22		−21%
2.142	77.09		−20%
3.559	128.1		−24%
5.488	197.5		−22%
*Electrode 3*			
*c*_pilot_	110		
*i*_pilot_	13.41		
0.2902	5.796	*see above*	−4.1%
0.5201	10.387		−14%
1.26	25.16		4.2%
2.322	46.37		−4.0%
4.896	97.78		1.3%
9.168	183.1		8.5%
13.39	267.4		5.8%
**August 20, 2010**			
*Electrode 1*			
*c*_pilot_	110.03		
*i*_pilot_	8.441		
0.3771	11.97	6.01	99%
0.4613	14.64	12.02	22%
0.8352	26.51	24.03	10%
1.711	54.30	48.03	13%
3.212	101.94	95.99	6.2%
5.402	171.4	167.8	2.2%
8.383	266.0	251.3	5.9%
*Electrode 2*			
*c*_pilot_	110.03		
*i*_pilot_	7.059		
0.3043	11.55	*see above*	92%
0.4153	15.76		31%
0.6217	23.59		−1.8%
1.062	40.30		−16%
2.142	81.29		−15%
3.559	135.1		−20%
5.488	208.3		−17%
*Electrode 3*			
*c*_pilot_	110.03		
*i*_pilot_	7.685		
0.3106	10.83	*see above*	80%
0.4024	14.03		17%
1.247	43.47		−9.5%
2.665	92.90		−3.2%
4.477	156.1		−7.0%
6.795	236.9		−5.7%
Average differences			
<15 microM			46%
>25 microM			13%
Overall			20%

**Figure 17:**
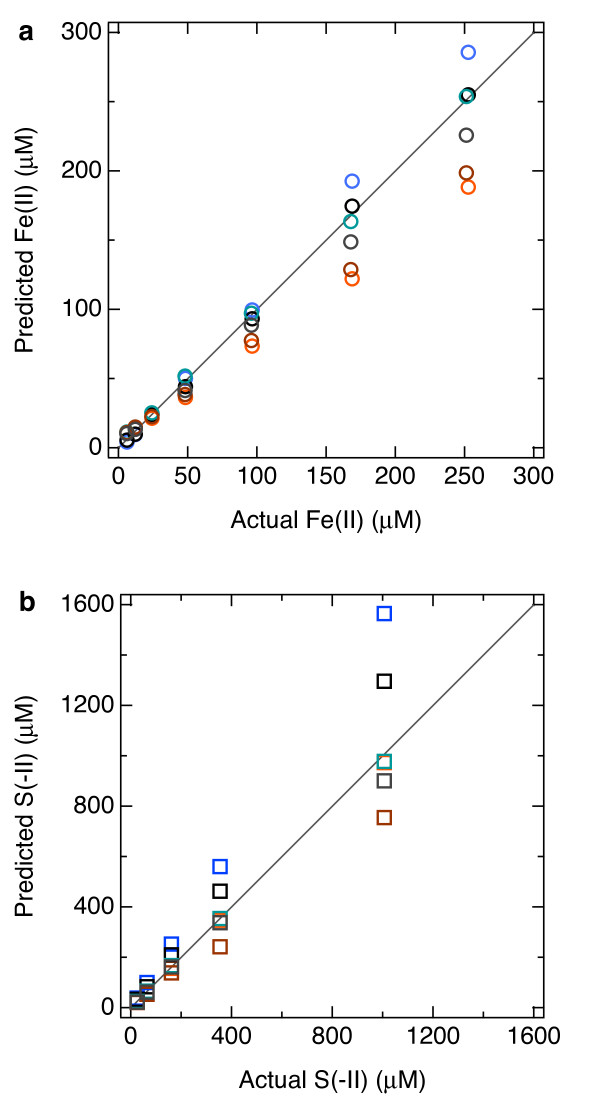
** Comparison of (a) Fe(II) and (b) S(−II) concentrations predicted by the pilot ion method where Mn(II) was the pilot versus their actual values.** The different colors indicate different days on which experiments were conducted, and related colors (turquoise and blue, brown and orange, and black and gray) indicate a common electrode, although the Hg/Au sensors were reconditioned as described in the text. The symbols are larger than the 90% confidence intervals

$c_{\text{u}}=2.43(1.71)\left(\frac{110}{8},.44\right)=54.3\;\mu \text{M}$ which is 13% higher than the actual concentration of 48.0 μM (Table 3). At 6 μM Fe(II), the discrepancy between concentrations predicted by the pilot ion method and their actual values was on average 46% and as high as 99% (Table 3). Fe(II) concentrations predicted by the pilot ion method are plotted versus their actual values in Figure 17a. That the pilot ion method seems to lose accuracy at lower concentrations has, to our knowledge, not been documented, and highlights the need to critically evaluate the evidence provided by Meites [28], which is limited to exaggerated concentrations of metals (hundreds of μM). That said, despite the fact that the seawater was acidified to pH 6 and maintained under a positive pressure of UHP N_2_, we cannot rule out oxidation of small amounts of Fe(II) as the cause of this discrepancy. S(−II) concentrations calculated using Mn(II) as the pilot ion and their actual values are listed in Table 4 and plotted against one another in Figure 17b. The S(−II) concentrations predicted by the pilot ion method were as much as 58% different from their actual values (21% on average for 29 data). On average, the discrepancy between predicted and actual S(−II) concentrations was twice that of Fe(II). Similarly, the 95% confidence interval for $K=s_{\text{Mn}}/s_{s\left(-\text{II}\right)}$ was nearly twice that of $\left(s_{\text{Mn}}/s_{\text{Fe}}\right)$ In conclusion, the accuracy of the pilot method is closely related to how independent *K* is from electrode to electrode.

**Table 4:** Comparison of actual S(-II) concentrations to those predicted by the pilot ion method

***K =*** **0.284**			
**i(Fe), nA**	**predicted*c***_**S(-II)**_**μM**	**actual*c***_**S(-II)**_	**Difference**
**August 19, 2010**			
*Electrode 1*			
*c*_pilot_	110		
*i*_pilot_	6.02		
7.404	38.37	25.4	51%
19.24	99.71	64.3	55%
48.68	252.3	161	57%
108.1	560.2	354	58%
302	1565	1006	56%
*Electrode 2*			
*c*_pilot_	110		
*i*_pilot_	7.441		
5.605	23.50	*see above*	−7.5%
14.64	61.38		−4.5%
37.26	156.2		−3.0%
82.76	347.0		−2.0%
232.1	973.1		−3.3%
*Electrode 3*			
*c*_pilot_	110		
*i*_pilot_	13.41		
14.38	33.45	*see above*	32%
35.68	83.01		29%
90.36	210.2		31%
198.8	462.5		31%
556.9	1296		29%
**August 20, 2010**			
*Electrode 1*			
*c*_pilot_	110.03		
*i*_pilot_	8.441		
6.752	24.96	25.4	−1.7%
17.72	65.51	64.3	1.9%
45.19	167.1	161	3.8%
95.74	353.9	354	0.0%
264.6	978.2	1006	−2.8%
*Electrode 2*			
*c*_pilot_	110.03		
*i*_pilot_	7.059		
4.69	20.73	*see above*	−18%
12.17	53.80		−16%
31.22	138.0		−14%
54.79	242.2		−32%
170.8	755.1		−25%
*Electrode 3*			
*c*_pilot_	110.03		
*i*_pilot_	7.685		
5.477	22.24	*see above*	−12%
15.24	61.88		−3.8%
39.8	161.6		0.4%
83.07	337.3		−4.7%
221.8	900.6		−10%
Average difference			21%

### Implications of different matrices

**Figure 18:**
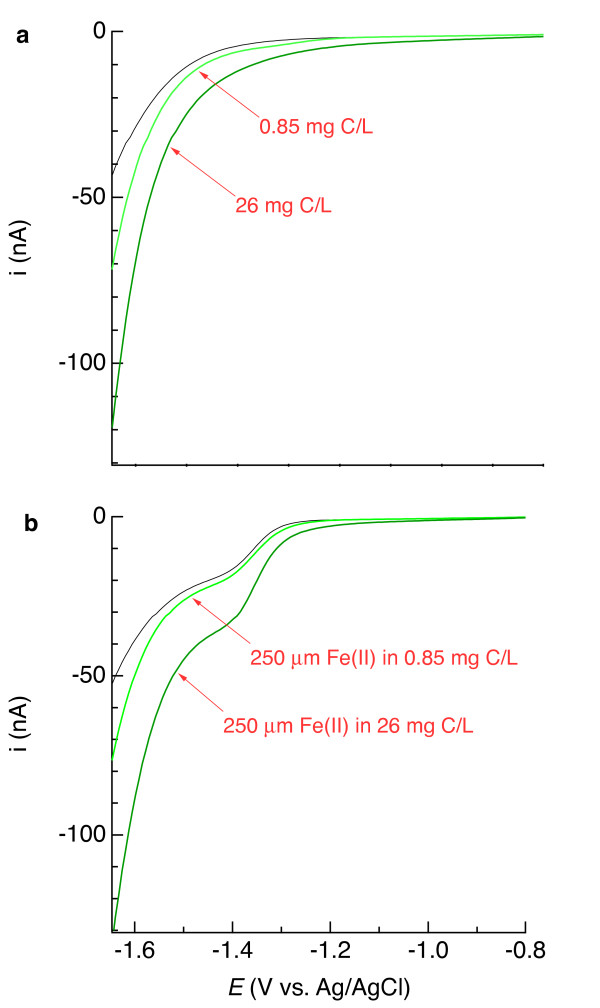
** (a) Current (*i*) versus potential (*E*) relationship in linear-sweep mode in artificial seawater (grey line) and artificial seawater containing 0.85 (mg C)/L Pacific Ocean fulvic acid (light green line) and 26 (mg C)/L Pony Lake fulvic acid (dark green line).** Panel b shows the same media amended with 250 μM Fe(II).

In most cases, we cannot or do not wish to chemically alter the medium we measure. Since the physicochemical composition of the medium can appreciably affect voltammetric signals [10], electrodes should ideally be calibrated in the same experimental test medium or natural water under investigation. Matching matrices may, however, be difficult. For instance, tidal flushing will change sediment pore water chemistry [16] and biological activity can change the properties of organic matter in sediment with depth [50]. Even if one extracted, for example, pore water from sediment to make standards in the laboratory, pore water chemistry will likely have changed by the time field measurements occur. What effect does the extent to which one matches the experimental matrix have on the accuracy of a voltammetric analysis? In the current study, DOC in artificial seawater noticeably changed the voltammetric currents throughout the Hg/Au polarization range (Figure 18). The examples provided in Figure 18 show how linear-sweep voltammetry provides evidence of matrix differences, and there are other voltammetric techniques that can more directly investigate the interactions between organic matter and electrodes [25,51]. Seawater standard solutions containing DOC were measured on different days than organic-free seawater. As a result, current response varied in part because of changes in electrodes’ intrinsic properties, as indicated earlier. However, the calibration slope ratio $K=\left(s_{\text{Mn}}/s_{\text{Fe}}\right)$ was found to be independent of electrode within a reasonable degree of variability. Closer examination reveals that the variance of *K* determined from analyses of DOC-free seawater was statistically indistinguishable from the variance of *K* determined from analyses of seawater amended with 0.850 (mg C)/L Pacific Ocean fulvic acid, on the basis of ANOVA and Bartlett’s test [52]. Despite appreciable changes in total signal (Figure 18), DOC at average-to-elevated oceanic concentration has an insignificant effect on the slope ratios of Mn(II) and Fe(II). Evidently, the concentration of DOC was too low to alter the electroactivity of Fe(II) and Mn(II), while 4^th^-order polynomials captured the change in nonfaradaic and faradaic (Na^+^) background current in the Fe(II) and Mn(II) potential region. Unlike Fe(II) and Mn(II), the presence of 0.850 (mg C)/L Pacific Ocean fulvic acid or 26 (mg C)/L Pony Lake fulvic acid in seawater did affect the slope ratio $K=s_{\text{Fe}}/s_{\text{s}\left(-\text{II}\right)}$, in that the variance of *K* from analyses of each DOC-amended seawater significantly differed at the 90% confidence level from that determined from organic-free seawater. This finding likely reflects a change in how HgS forms on the Hg/Au surface in the presence of DOC. DOC affects the kinetics of HgS precipitation, and spectroscopic analysis indicates that DOC also affects the molecular and colloidal properties of HgS [53]. In conclusion, matrix physicochemistry will likely affect the quantitative interpretation of voltammetric data, but one can determine whether baseline removal accounts for unavoidable differences in background currents measured in calibration and test solutions.

Temperature affects the diffusion of chemical species at the electrode-water interface, which can change the current by up to 10% per°C in the range 4–20°C [10,21]. If temperatures of lab or field systems are expected to differ from the temperature at which calibration data were obtained, the voltammetric figure of merit needs to be determined at a given species concentration as a function of temperature. The voltammetric figure of merit is typically significantly correlated to temperature for electrochemically reversible (HgS/Hg^0^) and irreversible (O_2_/H_2_O_2_, Fe^2+^/Fe^0^, and Mn^2+^/Mn^0^) systems and therefore can be used to correct *in situ* data [54,55]. The possible compensatory effect of lowered O_2_ and H_2_S solubility and higher rate of diffusion (and therefore larger diffusion-limited current) at higher temperatures should also be considered [13].

### Guidelines for baseline fitting

**Figure 19:**
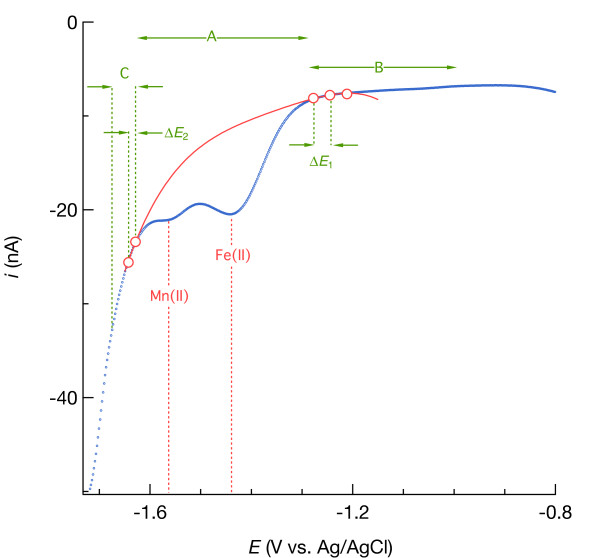
** Square-wave voltammogram of sediment from a Mississippi Gulf Coast marsh, indicating different regions for baseline function fitting, the chosen baseline points (anchors) through which the function must pass, and the electrical potential separation between adjacent anchors (Δ*E***).

To quantify electroactive constituents such as Fe(II), the faradaic current arising from electron transfer from the electrode to reduce Fe(II) to Fe(0) needs to be isolated from the nonfaradaic (capacitative) current plus any overlapping faradaic currents (e.g., from Mn(II) and Na^+^ reduction). As discussed earlier, we can favorably set the scan rate and use square-wave or other pulse techniques to eliminate some, but not all, of the capacitative current, but the remaining background needs to be removed. To do that, we fit an artificial function to data in the background portion of the signal immediately surrounding the region of interest (red curve in Figure 19) and then subtract the function. Fitting a 4^th^-order polynomial involves five degrees of freedom and, therefore, requires more user judgment than fitting a linear baseline. The importance of user judgment is underscored by the seeming invisibility of reduction peaks arising from Fe(II) and Mn(II) at concentrations of about 25 μM and less (Figure 6 and Figure 4). The following discussion raises aspects of nonlinear baseline fitting that the reader may find helpful to keep in mind.

With standard solution measurements, we fit baselines (see the Methods section), subtract them from the total signal, obtain *i*_p_ for the constituent of interest, plot *i*_p_ versus concentration, perform a linear regression, and evaluate the quality of the regression. Specifically, how well correlated is *i*_p_ to concentration? There should be a high degree of correlation (R^2^ > 0.99) if baseline fits were done correctly. The linear regressions should include computation of the confidence interval of the *i*_p_-axis intercept at zero concentration. This offset should be essentially zero if baseline removal was done correctly. But if we do not know the concentration, how do we know if the baseline removal was done correctly?

To start, we compare a sample voltammogram with that measured in standards to get a rough idea of the concentration. Points where the baseline function must intersect (anchors) are then chosen at potentials similar to those chosen with a standard voltammogram. If the overall polynomial fit looks unreasonable, the points are shifted slightly until the fit looks reasonable. By reasonable, we mean that the baseline follows the background signal on either side of the faradaic current region and does not exhibit strange topology, such as inflections within the faradaic region (region A in Figure 19). Furthermore, after subtracting the baseline, the symmetry and coincidence of peaks to the redox potential of Fe(II) or Mn(II) provide further indication of the quality of the baseline fit (e.g., Figure 6, Figure 4, and Figure 11).

Regions of voltammograms at potentials more positive than where Fe(II) and Mn(II) reduction occurs tended to overlap (region B in Figure 19; see also Figure 6 and Figure 4). Therefore, regardless of concentration, we chose three common baseline points in region B (Figure 19) and still achieved good correlations. However, the two remaining necessary anchors, chosen on the more negative side of the reduction signal (region C in Figure 19), need to be shifted by about +/−15 mV per halving/doubling of Fe(II) or Mn(II) concentration to follow the negative shift of the reduction signal as concentration increases (Figure 8). If anchors are chosen too far from the faradaic region, *i*_p_ will be overestimated. Good calibration curves were obtained when adjacent anchors were separated by about 20 mV. The distance Δ*E*_1_ in Figure 19 between the anchor nearest the more positive side of the reduction signal and the inflection point in the voltammogram indicating the onset of Fe(II) or Mn(II) reduction was typically 100 mV, while 40 mV typically separated the inflection point after the reduction signal and the nearest anchor on the negative side (Δ*E*_2_ in Figure 19).

As a sensitivity analysis, we adjusted the positions of anchors by 10 mV while maintaining what appeared to be a reasonable fit to the background signal. Such adjustment changed *i*_p_ by ±30% at the 1 nA level, which translates for our electrodes to uncertainties of 25 to 40 μM Fe(II) and approximately 20 μM Mn(II). At higher concentrations, it is easier to visually optimize the baseline fit, and we found that the uncertainty could be limited to 10% or less.

### Implications for redox characterization of aquatic systems

Voltammetric *i* – *E* relationships contain more information than potentiometric or amperometric signals, which enables the (nearly) simultaneous quantification of multiple redox constituents in minimally disturbed systems. In eliminating the need to repeatedly gather and process calibration data for every electrode used, the pilot ion method is useful for accurate redox characterization of aquatic systems with voltammetric solid-state electrodes. Aspects including electrode quality and data processing presented here will help new users more efficiently obtain quantitative results and understand their uncertainty. We hope this work will improve understanding of the exceptional capability of voltammetry to fill data gaps in understanding the economic and ecological function of terrestrial and aquatic systems.

## Competing interests

The authors declare that they have no competing interests.

## Authors’ contributions

AJS performed all analyses, experimented with the pilot ion method, identifying the steps necessary to carry it out as well as alternative approaches and their associated analytical uncertainties, and wrote this manuscript. MMD helped plan experiments and suggested revisions for the manuscript. All authors read and approved the final manuscript.

## Supplementary Material

Additional file 1 Example quantification of Fe(II) using Mn(II) as the pilot ion. Click here for file
