# Supplementary material for: How to overcome inter-electrode variability and instability to quantify dissolved oxygen, Fe(II), mn(II), and S(−II) in undisturbed soils and sediments using voltammetry
Source: Geochem Trans. 2012 Jun 25;13:6. doi: 10.1186/1467-4866-13-6 (PMC3442984; doi:10.1186/1467-4866-13-6)
Supplement: Additional file 1 — Example quantification of Fe(II) using Mn(II) as the pilot ion. [file 1467-4866-13-6-S1.pdf]

## Example quantification of Fe(II) using Mn(II) as the pilot ion

In an anaerobic chamber, prepare two replicate solution matrices similar to, if not matching, the medium in which unknown concentrations are to be determined. Most importantly, define the temperature, pH, and ionic strength of the solution. Prepare a fresh Fe(II) stock solution and a Mn(II) stock solution for addition to the matrix. Make several additions of Mn(II), performing a voltammetric scan after each addition, such as is described in the methods section. Make several additions of Fe(II) to the replicate solution, performing a voltammetric scan after each addition. For each concentration (*c*), determine the current (*i*) response and perform linear regressions on the (*c*,*i*) data to determine the slope (current/concentration) for both Mn(II) and Fe(II). Divide the slope of the pilot ion [Mn(II)] by the slope of Fe(II) to calculate the coefficient *K* in equation 3. At a later point and with any electrode, measure the current response of the electrode to the pilot ion $\left(i_{\mathrm{pilot}}\right)$ to a known concentration ($c_{\mathrm{pilot}}$), such as 100 μM of Mn(II), in the solution matrix. Then measure the unknown current response for Fe(II) in the sample $(i_{\mathrm{u}})$, multiply by *K* found in the anaerobic chamber experiments, and multiply by the response of the electrode to the pilot ion $\left(c_{\mathrm{pilot}}/i_{\mathrm{pilot}}\right)$, as with equation 3:

$$c_{\mathrm{Fe}} = K\ i_{\mathrm{Fe}} \left(\frac{C_{\mathrm{Mn}}}{i_{\mathrm{Mn}}}\right)$$
